# Area-Specific Information Processing in Prefrontal Cortex during a Probabilistic Inference Task: A Multivariate fMRI BOLD Time Series Analysis

**DOI:** 10.1371/journal.pone.0135424

**Published:** 2015-08-10

**Authors:** Charmaine Demanuele, Peter Kirsch, Christine Esslinger, Mathias Zink, Andreas Meyer-Lindenberg, Daniel Durstewitz

**Affiliations:** 1 Department of Theoretical Neuroscience, Bernstein Center for Computational Neuroscience, Psychiatry, Central Institute of Mental Health, Medical Faculty Mannheim, Heidelberg University, Mannheim, Germany; 2 Department of Psychiatry and Psychotherapy, Central Institute of Mental Health, Medical Faculty Mannheim, Heidelberg University, Mannheim, Germany; 3 Department of Clinical Psychology, Central Institute of Mental Health, Medical Faculty Mannheim, Heidelberg University, Mannheim, Germany; 4 Department of Neurology, Otto-von-Guericke-University, Magdeburg, Germany; Center for BrainHealth, University of Texas at Dallas, UNITED STATES

## Abstract

**Introduction:**

Discriminating spatiotemporal stages of information processing involved in complex cognitive processes remains a challenge for neuroscience. This is especially so in prefrontal cortex whose subregions, such as the dorsolateral prefrontal (DLPFC), anterior cingulate (ACC) and orbitofrontal (OFC) cortices are known to have differentiable roles in cognition. Yet it is much less clear how these subregions contribute to different cognitive processes required by a given task. To investigate this, we use functional MRI data recorded from a group of healthy adults during a “Jumping to Conclusions” probabilistic reasoning task.

**Methods:**

We used a novel approach combining multivariate test statistics with bootstrap-based procedures to discriminate between different task stages reflected in the fMRI blood oxygenation level dependent signal pattern and to unravel differences in task-related information encoded by these regions. Furthermore, we implemented a new feature extraction algorithm that selects voxels from any set of brain regions that are jointly maximally predictive about specific task stages.

**Results:**

Using both the multivariate statistics approach and the algorithm that searches for maximally informative voxels we show that during the Jumping to Conclusions task, the DLPFC and ACC contribute more to the decision making phase comprising the accumulation of evidence and probabilistic reasoning, while the OFC is more involved in choice evaluation and uncertainty feedback. Moreover, we show that in presumably non-task-related regions (temporal cortices) all information there was about task processing could be extracted from just one voxel (indicating the unspecific nature of that information), while for prefrontal areas a wider multivariate pattern of activity was maximally informative.

**Conclusions/Significance:**

We present a new approach to reveal the different roles of brain regions during the processing of one task from multivariate activity patterns measured by fMRI. This method can be a valuable tool to assess how area-specific processing is altered in psychiatric disorders such as schizophrenia, and in healthy subjects carrying different genetic polymorphisms.

## Introduction

Most experiments in cognitive neuroimaging try to understand cortical function through subtraction and task designs that isolate specific cognitive functions. This approach is limited because the same brain region may contribute in diverse ways over the course of task performance. Identifying these different contributions over time can illuminate different stages of cognitive processing and thereby enhance our understanding of the computations occurring in specific regions. New methodological approaches are necessary to disentangle the diverse contributions of a given brain region to a task and to identify how regions differ in their processing, instead of just ascertaining their general involvement. The present study applies such methods to understand the role of subdivisions of prefrontal cortex (PFC) to probabilistic inference.

The prefrontal cortex (PFC) plays a major role in behavioral organization and decision making [1–3], and both animal work [4–6] and human neuroimaging studies [7,8] have provided evidence for a differential role of the various subdivisions of the PFC in these processes: The dorsolateral prefrontal cortex (DLPFC) is prominent in functions such as working memory, prediction of future outcomes, rule-based behavior, and planning and problem solving [9–11]. The anterior cingulate cortex (ACC) is, instead, believed to be more involved in response monitoring [12], in the integration of outcomes, and potentially in guiding choices (see Walton et al., [13] for review; [6,8,14]. On the other hand, the orbitofrontal cortex (OFC) is more involved in reward processing [15,16], and the actual encoding of stimulus and action values [17,18]. These previous studies, however, dissociated the role of PFC subregions either by employing different tasks or by focusing on just one brain area at a time in animal experiments [4–6]. Only a few studies have attempted to differentiate the contribution of PFC regions within one and the same experimental condition [19].

In this work, we reanalyzed a subset of previously published fMRI blood oxygenation level dependent (BOLD) data recorded from a group of healthy adults during a Jumping to Conclusions (JTC) probabilistic decision making task in order to assess differential contributions of the DLPFC, ACC, and OFC to task performance [20]. This task is a modified version of the “beads task” during which participants iteratively gathered evidence about two choices before they reached a final decision and then rated how confident they were about their decision. Esslinger et al. [20] performed a hybrid analysis on this data, combining both block design and event-related approaches, and revealed a tonic-activation of prefrontal cortex during the decision making process. However, while this previous study established that these areas were activated, it did not differentiate between their specific information processing roles during the task. Here we apply multivariate statistical and machine learning tools, including a feature selection algorithm, to investigate in more detail the different aspects of decision making processing taking place in the DLPFC, ACC, and OFC. Multivariate methods aim to extract the information contained in *patterns* of activation that form the multivariate fMRI BOLD time series [21–25] and have been employed in previous neuroimaging studies to track decision making and higher-level cognitive processes [19,24,26–29]. Although the analysis methods employed here build upon both classical [30,31] and state of the art [32] statistical procedures, to the best of our knowledge they have not been previously employed on fMRI BOLD data and used to gain insight into the stages of probabilistic reasoning in specific brain areas.

## Materials and Methods

### Ethics

The study was approved by the local ethics committee of the Medical Faculty Mannheim of the University of Heidelberg (AZ 2009-296N-MA) in accordance with the Declaration of Helsinki. Participants received written and oral instructions of the procedures, and gave written informed consent.

### Task

The experimental design is described in detail in [20]. In short, subjects viewed fish of two colors jumping out of a lake and had to decide from which of two lakes they were coming. The two possible source lakes contained fixed (80/20% or 20/80%) fish-colour ratios, and explicit information on the fixed ratios was provided to the participants. The colored fish were presented in a previously defined sequence (e.g. lake 1-1-1-2-1-1-1-1-2-1), recapitulated eight times with alternative starting points to avoid stereotypical responses to all trials. After each fish, subjects were asked if they wanted to see another fish. Subjects were told they could view as many fish as they wanted without any pressure on speed or accuracy. However, for methodological reasons, the number of fish per block was restricted to ten. The previously chosen fish were maintained on the screen. After presentation of the selected number of fish or a maximum of ten fish, subjects had to choose one lake and then rate on a four-point scale how confident they were about their decision (1 = a little uncertain and 4 = totally certain). In the control condition that followed, which required similar visual and motor demands, subjects had to only indicate the color of presented fish. The whole experiment consisted of eight trials of 2.2 minutes each; each trial comprised one JTC block and one control block. The analyses presented here were conducted on the 8 JTC blocks.

### Data acquisition and preprocessing

BOLD fMRI was performed on a 3T Siemens Trio by using gradient echo, echo-planar imaging (28 axial slices; 4mm thickness; 1mm gap; TR/TE 2,000/28 ms; flip angle 80°; FOV 19.2 cm; matrix 64 × 64; 526 scans). The datasets underwent a typical fMRI pre-processing stream, including slice time correction, realignment and spatial normalization to a standard EPI template, using SPM8 (Wellcome Department of Imaging Neuroscience, Institute of Neurology, London, UK). The first four volumes of each run were discarded to account for magnetic saturation effects. No spatial smoothing was applied. Data were standardized (i.e. centered and scaled to have zero mean and unit variance), detrended and high pass filtered at 256 seconds.

Voxel time points were labeled according to six task epochs associated with the 1) *first*, 2) *second*, 3) *third* and 4) *fourth* fish drawn (representing the iterative accumulation of evidence), with 5) *behavioral response*, and with 6) *choice-rating*.

From the whole dataset, a subsample of 11 from the 13 participants who performed the classical version of the task [20] and had a mean draws-to-decision of four fish was selected for our multivariate analysis. One female was excluded because she viewed all 10 fish during five of the eight blocks (this dataset was excluded also from [20]). The other male participant was excluded for having a mean fish draw of 2.25, (2 trials with 3 draws, and no trials with 4 draws), which meant that there would not be sufficient data points to properly represent the accumulation of evidence phase of the task for this participant. The remaining sub-sample of 11 participants provided us with a dataset that did not have performance differences as a potential confound, facilitated even segmenting of task stages, ensured similar number of data points in each class, and facilitated comparisons across participants.

Analyses were conducted on ROIs defined according to the Harvard-Oxford probabilistic mask atlas (http://fsl.fmrib.ox.ac.uk/fsl/fslwiki/Atlases/), thresholded at 50%. In this work we were interested in probing our novel methodological approaches specifically to distinguish between the information processing roles of PFC subregions, namely the DLPFC, ACC and OFC during the JTC, since they are well known to play differential roles in decision making, because of their special relevance to schizophrenia [33–35] and because of a larger body of animal work to compare our results to [14,15,36–38]. Thus, we mainly considered the DLPFC, ACC and OFC as task-related ROIs. We also included the hippocampus due to its well-documented association with the DLPFC [39,40]. Furthermore, we considered non-task-related regions, mainly the anterior temporal fusiform cortex (TFC) and the inferior temporal gyrus (ITG), as our control regions since for these areas there is little or no evidence for a prominent contribution to core processes of decision making, and hence they could serve in validating our approach.

### Multivariate test statistics

To investigate the contribution of prefrontal (DLPFC, ACC, and OFC) and temporal (non-task-related, control) regions to information processing in the JTC task, we first grouped the whole time on task into segments corresponding to the six task stages associated with the *first*, *second*, *third* and *fourth* (last) fish drawn before decision (these classes represent the iterative accumulation of evidence), with *behavioral response*, and with *choice-rating* (i.e. a total of six stages/classes). Three multiple-group multivariate test statistics were computed separately for each ROI, namely Roy’s greatest characteristic root (GCR), Wilk's lambda, and Hotelling's Generalized T^2^ [30,31]. These test statistics provide a measure of the overall discriminability of the different task stages, and thus of the amount of information contained within the multivariate voxel patterns about these stages. Furthermore, pairwise differences between two specific task stages were computed by means of Mahalanobis distances [30].

**Fig 1:**
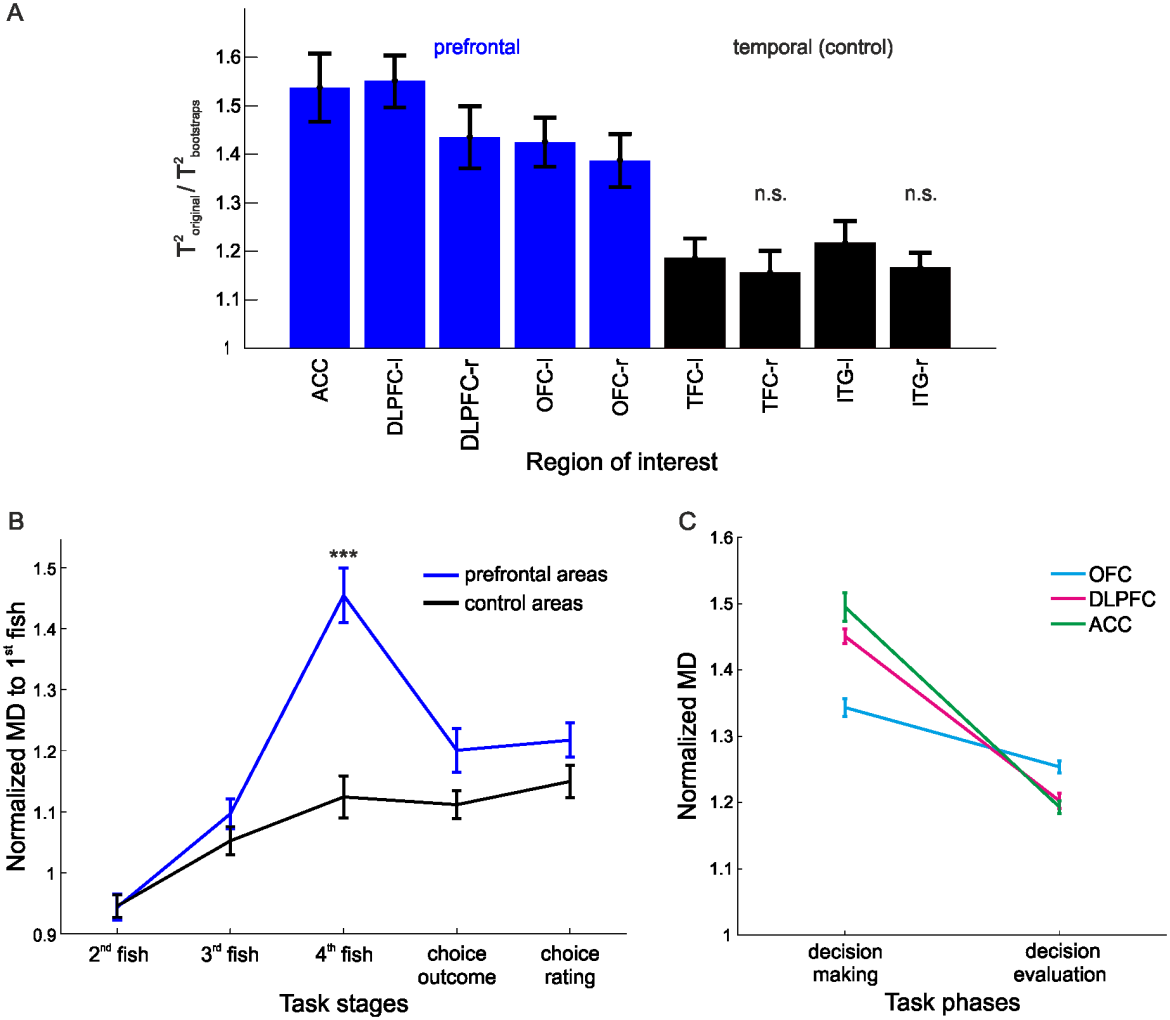
Multivariate test statistics. (*A*) Hotelling’s Generalized T^2^ for prefrontal (blue) and non-task-related (black) temporal regions normalized by the corresponding bootstraps; n.s. = not significant w.r.t. bootstraps. (*B*) Mahalanobis distances (MDs) to the first fish drawn before decision (normalized w.r.t. bootstraps) clearly revealed a distinct "decision" point, which was significantly more pronounced in prefrontal regions (blue). (*C*) Differences in normalized Mahalanobis distances during the *decision making* and the *decision evaluation* phases for the ACC, DLPFC and OFC; N = 11; Error bars = SEM across participants.

Since time series data violate the independence assumption required for conventional general-linear-model-based parametric statistical testing, non-parametric bootstraps were used to test the significance of classification results. Bootstraps were constructed by shuffling, for each trial, whole blocks of identical consecutive class labels corresponding to a given task stage hence preserving the autocorrelations within the original data. To account for the variable number of voxels in the different ROIs, for every ROI, the test statistics and their corresponding bootstraps were computed 50 times with *v* voxels chosen at random from the voxels within that ROI, where *v* is the total number of voxels within the smallest ROI. The average value was computed as the test statistic for that ROI, and tested for significance against the bootstrap distribution (comprising 1000 bootstrap values, each representing an average across 50 draws as defined above). This procedure was repeated for every participant, and the average across participants is shown in Fig 1*A*. Mahalanobis distances were statistically verified in the same manner. All analyses were computed on the preprocessed time series with the behavioral labels shifted by 2 TRs in order to compensate for the hemodynamic lag; this optimal lag was determined by computing the canonical correlation between the BOLD time series and the behavioral labels [41].

### Searching for sets of most predictive voxels

We adapted a variable selection method, which builds on the algorithm presented in [32], and modified it for use on fMRI data to select those voxels from some set of ROIs which contain maximal information about specific experimental conditions. The method builds on a linear discriminant analysis (LDA)-classifier *C* and leave-one-out cross validation [42], and attempts to find the maximally predictive set of voxels through a forward-backward procedure. For any particular set of selected voxels, a test data point *x*_*i*_ (i.e., activity pattern across the set of selected voxels) is assigned to the class *k* to which it has minimum Mahalanobis distance $\delta_{k}(x_{i})=\sqrt{\left(x_{i}-\mu_{k}\right){\hat{\sum }}^{-1}{\left(x_{i}-\mu_{k}\right)}^{T}}$, where $\hat{\sum }=\left(1-\lambda \right)\sum +\lambda \text{diag}\left(\sum \right)$ is the regularized pooled covariance matrix; thus classification of *x*_*i*_ is given by $C\left(x_{i}\right)=\underset{k}{\text{argmin}}\delta_{k}\left(x_{i}\right)$. The average cross-validation error (CVE) for a set of voxels *S* is then defined as $CVE\left(S\right)=\frac{1}{n}\sum_{i}I\left[C_{LDA}^{-i}\left(x_{i}\right)=C_{true}\left(x_{i}\right)\right]$, where *n* is the number of time points, *I* is the indicator function, superscript-*i* indicates that data point *x*_*i*_ was left out for building the discriminant classifier *C*_*LDA*_, and *C*_*true*_ returns the true class membership of *x*_*i*_. The forward-addition/ backward-deletion algorithm (described in detail in [32]) then proceeds by adding to the current voxel set *S*_*m*_ the voxel which leads to the maximum reduction in *CVE*(*S*_m+1_). It continues adding voxels until the penalized cross-validation reaches a minimum *PCV*(*S*_*m*_) ≥ *PCV*(*S*_m−1_), with *PCV* = *CVE* × (1 + ξ*p*/log*n*), where *p* is the number of voxels and ξ is a constant (here set to 0.1). In the backward loop, voxels are then eliminated again as long as the *PCV* is still decreasing or remaining constant, i.e. until *PCV*(*S*_*m*_) > *PCV*(*S*_m+1_).

## Results

### Multivariate analysis of task-related information in different ROIs

We employed multivariate test statistics as defined for multivariate general linear models [31] to investigate the overall discriminability of these different task stages within the BOLD signal pattern for our ROIs. Test statistics were normalized by the same quantities computed from block permutation bootstraps to account for regional-specific differences in the (auto-) correlative structure of the multivariate BOLD time series (which were preserved in the bootstrap data; see Methods). Group results for Hotelling’s Generalized T^2^ normalized by the corresponding bootstraps are shown in Fig 1*A* (other multivariate statistics, Wilk’s Λ and Roy’s GCR, gave similar results). All ROIs with the exception of two non-task-related control regions achieved significance (at p < .05) when their T^2^ values averaged across participants were compared to their respective bootstrap distributions (cf. Fig 1*A*). This indicated that in fact most investigated brain areas contained a significant amount of information about the task. Furthermore, for an average of 8 out of 11 (range 7 to 10) participants, prefrontal areas had significant test-statistics with respect to their bootstrap distributions derived separately for each participant. This demonstrates that significance of task-related information could often be established at the individual, single-subject level. On the other hand, non-task-related areas achieved significance for an average of 3/11 (range 2 to 5) participants. Using the binomial distribution, this pattern was overall significant across all participants for prefrontal regions (*p*<10^−6^). Moreover, repeated measures ANOVAs (computed on the two grouped sets of ROIs shown in Fig 1*A*) revealed that prefrontal areas contained significantly more information about task structure (as measured by the normalized multivariate statistics) than temporal (non-task-related) regions (*F*(1,10) = 177.4, *p*<10^−6^).

Next, to investigate task-stage-specific activity patterns and regional differences in more detail, differences between any pair of task stages were quantified through Mahalanobis distances [30], normalized to bootstraps to compensate for time series autocorrelations and different temporal separation between task stages. A 2-factor repeated measures ANOVA (performed on the mean values across participants) revealed that the Mahalanobis distances from the presentation of the first fish drawn before decision to the other task stages significantly differed between prefrontal and control regions (*F*(1,10) = 32.85, *p* < .001) and across task stages (*F*(4,7) = 35.32, *p* < .001; Fig 1*B*). Importantly, the interaction effect (brain region x task stage) was also significant (*F*(4,7) = 17.92, *p* < .001), likely because Mahalanobis distances culminated at the last choice, right before the behavioral response, for prefrontal but not for control regions (Fig 1*B*). This was further supported by a significant linear trend across the first three task stages towards the decision point (i.e., presentation of the fourth (last) fish; *F*(1,10) = 92, *p*<10^−6^). This effect is unlikely to be due to the closer temporal proximity (i.e. temporal autocorrelations) between the last and immediately preceding fish presentations compared to those earlier in the sequence since a) the effect was much weaker in control regions (*F*(1,10) = 20.1, *p*<0.001), b) Mahalanobis distances were normalized to bootstraps with the same temporal spacing, and c) Mahalanobis distances declined again for later task stages despite the even wider temporal spacing. Thus, there was a gradual build-up of BOLD pattern separation during the process of evidence sampling up to the point where subjects made a decision. Test statistics and Mahalanobis distance results for the hippocampus and midbrain regions (ventral tegmental area, dorsal and ventral striatum) can be found in the Supporting Information (S1 File).

Considering the pattern of pairwise differences among task stages suggested that prefrontal ROIs, particularly the DLPFC, OFC and ACC, may be differentially involved in encoding aspects of the task. Fig 1*C* shows pairwise distances during the *decision making phase* (measured as Mahalanobis distances between the first, second and third to the fourth (last) fish before behavioral response) and during the *decision evaluation phase* (measured as pairwise distances between first, second and third fish to the behavioral-response and choice-rating stage). A repeated two-way ANOVA with ROI (ACC, OFC) and task phase (*decision making*, *decision evaluation*) as factors revealed a significant region x task-phase interaction (*F*(1,10) = 20.38, *p* < .001), as well as a significant main effect for task phase (*F*(1,10) = 13.5, *p* < .004). Similar results were obtained when the DLPFC and OFC were compared (region x task-phase interaction: *F*(1,10) = 10.13, *p* < .01; task phase: *F*(1,10) = 20.59, *p* < .001). In order to investigate the nature of the interaction, we compared the regions' mean Mahalanobis distances for the two task phases. Paired t-tests, Bonferroni corrected for multiple comparisons (significance at p = .05/2 to correct for the 2 comparisons being made), revealed that Mahalanobis distances for the ACC were significantly higher during decision making (t(1,10) = 2.9, p < .02) and significantly lower during decision evaluation (t(1,10) = -2.76, p < .02) when compared to those for the OFC. A similar relationship was observed for the DLPFC vs. OFC: DLPFC showed a trend towards higher Mahalanobis distances for decision making (t(1,10) = 2.51, p < .03) and lower distances for decision evaluation (t(1,10) = -2.12, p < .06) in comparison to the OFC. These data suggest that while the ACC and DLPFC are more involved in the actual decision making process, the OFC plays a larger role in evaluating the decisions.

### Extracting voxel subsets with highest task-stage predictability

**Fig 2:**
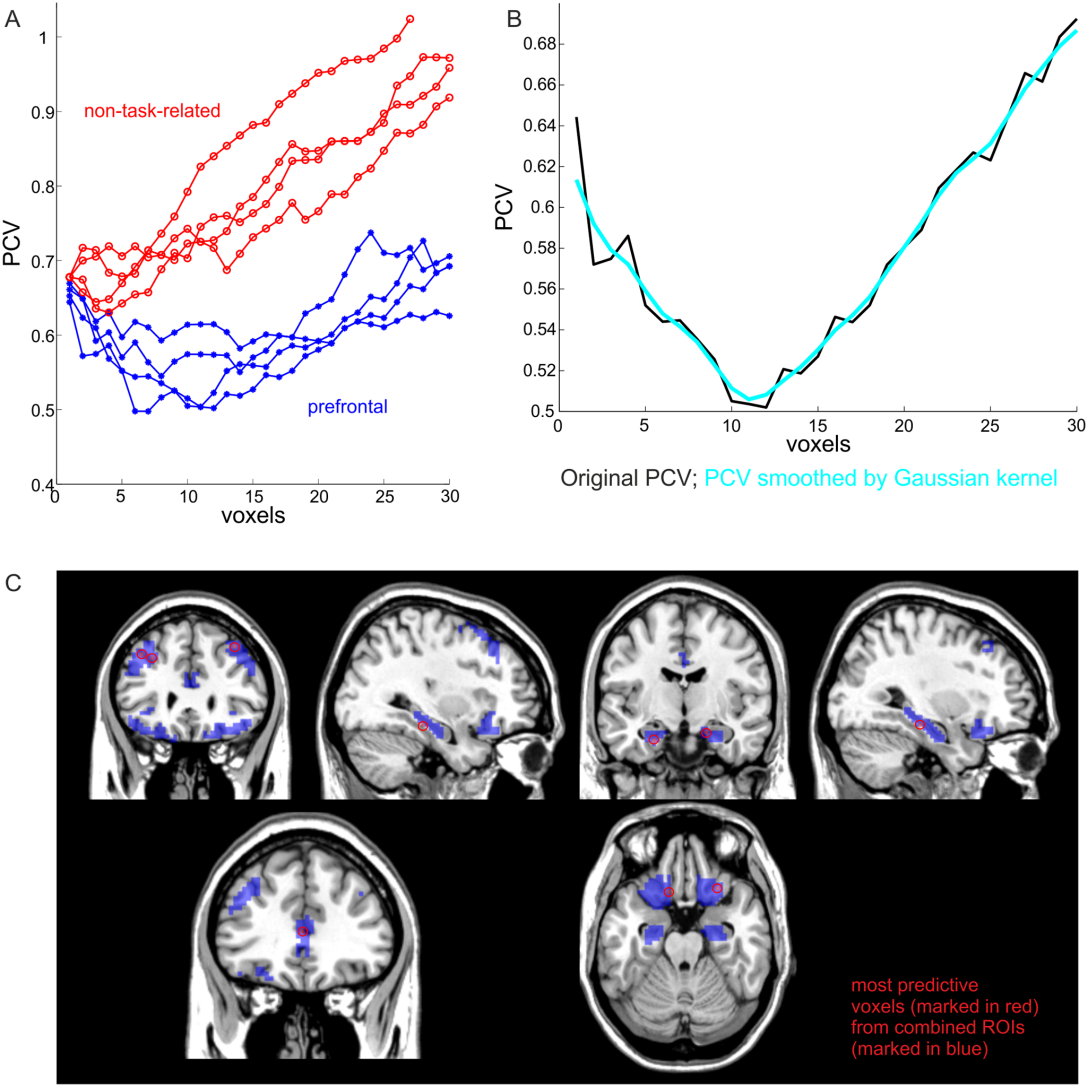
Voxel selection algorithm. (*A*) Penalized cross validation error for task-related (blue) and non-task-related (red) ROIs. (*B*) Penalized prediction error curve for the left DLPFC, smoothed by a Gaussian kernel (cyan). A minimum (non-penalized) prediction error of 0.327 = 0.505/(1 + ξ*p*/log*n*) was obtained for the overall separation of the six cognitive task stages; *p* is the number of voxels for this ROI (left DLPFC), *n* is the number of time points, and ξ = 1. (*C*) The most predictive voxels (marked in red) selected from a combined ROI mask (blue regions) were distributed across the DLPFC, OFC, ACC and hippocampus.

Rather than comparing the information content about various task stages between *a-priori* defined brain regions, in an alternative approach we wanted to identify those voxels from a set of regions that are most predictive about task stages, without pre-labelling voxels according to the areas they come from. Our algorithm (see Methods) identifies a subset of voxels that are most informative about specific task stages by measuring a form of prediction error (the penalized cross-validation error, PCV). Fig 2*A* shows separate PCV error curves for prefrontal (blue) and non-task-related (red) sets of ROIs. While the PCV error for prefrontal regions reaches a global minimum at about 10 voxels, the PCV error for non-task-related (temporal) regions is higher and continues to increase as more voxels are added (Fig 2*B* illustrates how minima were found in these curves by Gaussian kernel smoothing). Thus, while for prefrontal regions a set of ~10 voxels was optimal for predicting task stages from BOLD patterns, for non-task-related regions prediction was bad to begin with, and further deteriorated as more voxels were added, indicating that for those regions, patterns of activation did not really contain more information than single voxels. For prefrontal regions, a prediction error of only ~32.7% was obtained for the overall separation of the six cognitive task stages, compared to ~83.3% expected by chance. Furthermore, when the DLPFC, OFC, ACC and the hippocampus were combined into one mask and passed as a whole through this algorithm, the set of most predictive voxels was found to be distributed across all of these regions, demonstrating that they significantly contributed to information processing during the JTC task execution (Fig 2*C*).

**Fig 3:**
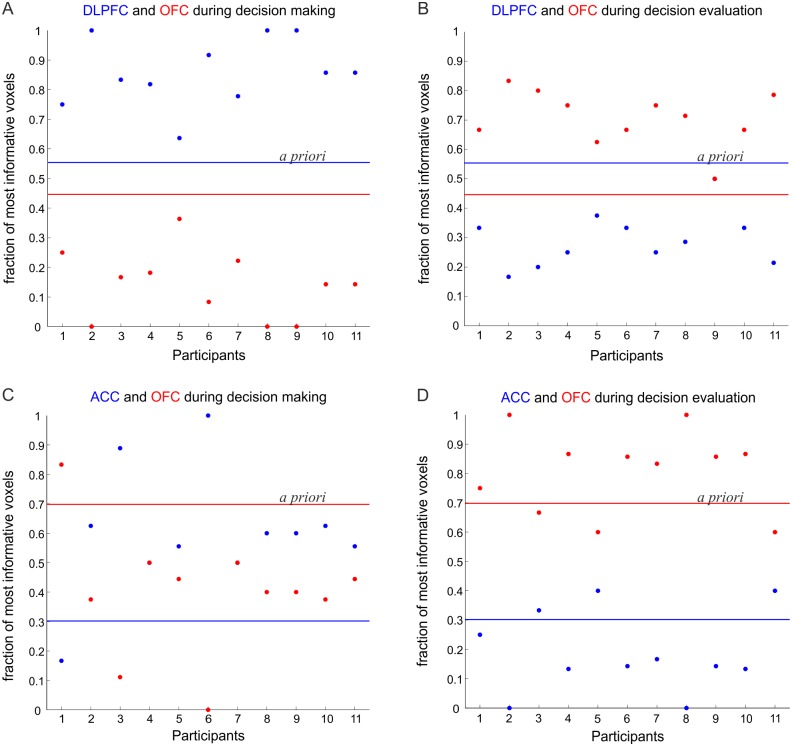
PFC subregions comparison. The fraction of most informative voxels selected during the (*A*) *decision making* and (*B*) *decision evaluation* phases from the DLFPC and the OFC for all of the 11 participants. (*C*) The fraction of most informative voxels selected during *decision making* and (*D*) *decision evaluation* phases from the ACC and the OFC for all participants. The blue and red lines represent the priors for the DLPFC/ACC and OFC masks, respectively.

We next used this algorithm to substantiate the findings obtained from the multivariate test statistics (shown in Fig 1*C*) with regards to the differential roles of PFC regions. We considered one region that was more involved in the decision making process (i.e. the DLPFC or the ACC) and compared it to the OFC region, which was more involved in evaluating the decisions. All OFC and DLPFC voxels were combined, and the voxel-selection algorithm was employed on this combined set. From the set of most-informative voxels extracted by our selection procedure from the combined mask, we then determined the proportion of voxels coming from the OFC vs. the proportion of voxels coming from the DLPFC for a) the *decision making* phase, defined by the first to third fish presentations preceding choice (class 1) vs. the fourth (last) fish presentation preceding choice (class 2), and b) the *decision evaluation phase*, defined by class 1 as above vs. the time points corresponding to the behavioral response and choice-rating (class 2). Thus, the first grouping contrasts the accumulation of evidence with the actual decision. The second grouping contrasts the accumulation of evidence with the evaluation of the decision outcome. Fig 3*A* shows the proportion of most informative voxels (posterior probabilities) selected from the DLFPC and the OFC for each participant during the decision process, together with the *a priori* probabilities of selecting voxels from the two areas (blue and red lines for DLPFC and OFC, respectively) due to the fact that the two masks contained a different number of voxels (DLPFC = 643, OFC = 518). During *decision making*, across all participants, using a sign (binomial) test the posterior probabilities (proportions of selected voxels) divided by the corresponding priors were significantly more often (in fact consistently across all participants) higher for the DLPFC than for the OFC (*p* < .001; Fig 3*A*). In contrast, during *decision evaluation* the OFC contained higher proportions (corrected to the priors) of selective voxels consistently across all participants (*p* < .001; Fig 3*B*). Similar results were observed when comparing the ACC to the OFC, with ACC > OFC across all participants during *decision making* (*p* < .02; Fig 3*C*), whilst OFC > ACC during *decision evaluation* (*p* < .03; Fig 3*D*). Thus, in agreement with the multivariate results from the previous section, the unbiased (by the experimenter choice of ROIs) voxel selection algorithm confirmed differential information processing roles for the DLPFC/ ACC and OFC during the JTC task.

## Discussion

In this work we brought a novel combination of multivariate statistical techniques to differentiate the timing and contributions of prefrontal cortical regions (DLPFC, ACC and OFC) to probabilistic inference using the BOLD response [20]. The task required participants to iteratively sample evidence (fish colors) that would inform a binary decision (which pond) based on probabilistic information. This involved integrating evidence across presentations, a function most closely associated with the ACC [13,14,36,43], up to the point that a decision threshold is reached and a response is triggered, possibly reflecting the role of the DLPFC [9,11,37]. Finally, subjects would have to rate their certainty for each response, a function most tightly linked to the OFC [7,15,16]. While all three prefrontal regions contained a significant amount of information about the different task stages in their BOLD activity pattern, and significantly more so than non-task-related areas, there were also informative differences. First, differences between BOLD activity patterns steadily increased across stimulus presentations up to the actual decision point in prefrontal regions. We suggest that this gradual increase across presentations may reflect the process of accumulation of evidence, similar to the gradual changes in single neuron firing rates that have been observed during evidence integration in primate cortex [44]. Second, there was a clear interaction, verified by two different approaches, between prefrontal brain regions and task stages: While BOLD patterns in the ACC and DLPFC contained more information about the initial evidence accumulation and decision phase, the OFC contained more information about the outcome evaluation and uncertainty rating, in line with the literature [7,15,16].

To reveal these results we used various multivariate test statistics, in combination with bootstrap distributions that shuffled blocks of task-stage labels and thus preserved auto-and cross-correlative properties of the original time series. In contrast to conventional univariate analysis, multivariate methods harvest the whole spatial pattern inherent in fMRI data, and hence may provide more information about task-related neural representations and processing [21,23,27]. The idea that multivariate patterns of BOLD activity contain substantially more information about task stages than the best discriminating single voxels was explicitly confirmed by our voxel selection procedure: Task stage prediction as measured by the cross-validation error improved as more and more voxels were added to the predictor set, up to a size of about 10 in task-related regions, even though the cross-validation criterion contained a further term penalizing for the number of predictors in the set. Interestingly, this was in strong contrast to the presumably non-task-related temporal regions, which in general also contained a significant amount of information about the task as compared to bootstraps, but which according to our variable selection method could mostly be condensed within just one properly chosen voxel. This result, as well as the fact that the profile of pairwise task-stage comparisons was rather flat in these areas (cf. Fig 1*B*), may suggest that some regions like the temporal cortices are not directly involved in detailed task-related information processing, but may inherit their informative properties from prefrontal top-down inputs or rather from general, brain-wide fluctuations in BOLD activity across the task.

Our results are in line with mounting evidence suggesting that the DLPFC, ACC, and OFC play interdependent, yet separable roles in decision-making. The DLPFC is believed to subserve executive functions, like the maintenance and manipulation of goal-relevant information in working memory [9,38,45]. It has also been implicated in different forms of deliberation and reasoning, including inductive reasoning [11,46], and prediction of future outcomes to guide choices [3,9,10,47]. Recent combined fMRI and repetitive transcranial magnetic stimulation (rTMS) studies also have provided causal links between disruption of DLPFC activity and decision making [11,48,49]. Evidence from primates involving ACC lesions and from neuroimaging studies in humans suggests that apart from its role in response monitoring and in encoding and integrating the reward value of actions based on the history of response-outcome associations [5,13,43,50,51], it also plays an active role in rule-based decision-making [4,6,29,52]. Finally, numerous human neuroimaging studies [7,16,53,54] as well as OFC lesions studies in humans [46,51,52] have suggested a role in outcome evaluation. Primate in vivo electrophysiological studies furthermore confirm that OFC neurons encode predicted and relative reward value and magnitude of options [6,15,55].

An important aspect of our work is that, in contrast to most previous applications of multivariate classifier techniques in fMRI research [19,23,27], here the different instances of the task stages (classes) to be discriminated were not widely separated in time; they were not from separate trials with larger temporal gaps between them. Although such designs are advantageous for discriminating and thus also predicting different task conditions or mental states from BOLD activity patterns, they do not easily allow the processing steps to be teased apart during continuously evolving, temporally extended tasks like the present one, and do not reflect the temporal dynamics of cognition unfolding in real time. Here we demonstrated that by using time series bootstrap techniques even task stages following closely in time can be statistically discriminated, with predictive power as evidenced by our cross-validation-based voxel selection procedure. This suggests that despite the considerable low-pass filtering properties of the fMRI signal, useful information about different temporally consecutive processing steps and their association with different brain areas can still be obtained.

Finally, on a more methodological issue, we would like to point out that the voxel selection algorithm would, by definition, find the maximally informative voxels from *any* set of voxels, brain areas, or ROIs provided as input to the algorithm. The algorithm itself is purely bottom-up driven and as such unbiased by, and agnostic to, any specific hypotheses the investigator may have in mind. However, due to the step-wise leave-one-out cross validation procedure, it would be computationally quite expensive to run on the whole brain, apart from the scientific and statistical benefits of narrowing down the search space to a specific set of derived hypotheses. Even when restricted to a specific set of ROIs, however, the bottom-up and multivariate nature of the algorithm could still help to pin down distribution or localization of function more specifically, or to differentiate more general (low-dimensional feature space) from more specific (higher-dimensional feature space) contributions of an area to information processing. Such finer-grained methods could reveal more subtle differences in regional information processing between subjects with different genetic or psychiatric backgrounds, bearing the potential for novel diagnostic tools or biomarkers.

In summary, our work provides evidence for the role of the DLPFC and ACC during the accumulation of evidence and probabilistic reasoning, and the role of the OFC in processing choice evaluation. It illustrates the power of new techniques to reveal intricate information inherent in fMRI data, illuminate the temporal unfolding of cognitive processing, and distinguish between the complementary roles of brain regions during one task.

## Supporting Information

S1 File Multivariate test statistics. (DOCX)
