# Supplementary material for: Area-Specific Information Processing in Prefrontal Cortex during a Probabilistic Inference Task: A Multivariate fMRI BOLD Time Series Analysis
Source: PLoS One. 2015 Aug 10;10(8):e0135424. doi: 10.1371/journal.pone.0135424 (PMC4530897; doi:10.1371/journal.pone.0135424)
Supplement: S1 File — (DOCX) [file pone.0135424.s001.docx]

**Supplementary Information**

**
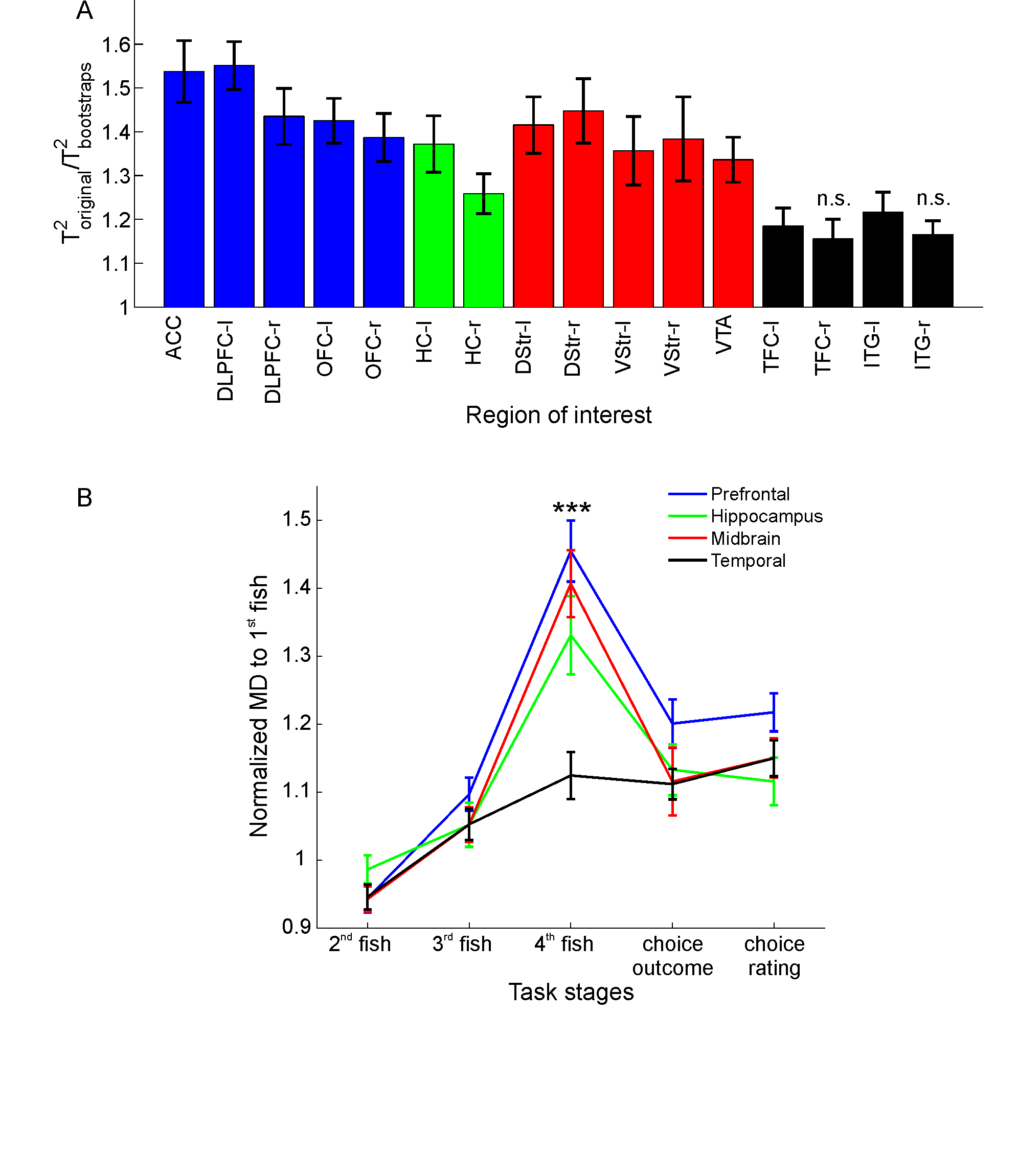
**

**S1 Fig. Multivariate test statistics.** (*A*) Hotelling’s Generalized T^2^ for prefrontal (blue), hippocampus (green), midbrain (red) and temporal control (black) regions normalized by their corresponding bootstraps; n.s. = not significant w.r.t. bootstraps. (*B*) Mahalanobis distances (MDs) to the first fish before decision (normalized w.r.t. bootstraps) clearly revealed a distinct "decision" point, which was significantly more pronounced in prefrontal regions, as well as in midbrain (VTA, dorsal and ventral striatum) and hippocampal regions, as opposed to temporal (control) regions.

With reference to S1 Fig., the ventral tegmental area (VTA) mask was constructed by drawing a region of interest on MRI-based anatomy of the VTA region using an anatomical atlas. For the dorsal (DStr) and ventral (VStr) striatum the WFU PickAtlas was used [1,2].

**Supplementary References**

1. Maldjian JA, Laurienti PJ, Kraft RA, Burdette JH. An automated method for neuroanatomic and cytoarchitectonic atlas-based interrogation of fMRI data sets. NeuroImage. 2003;19: 1233–1239. doi:10.1016/S1053-8119(03)00169-1

2. Maldjian JA, Laurienti PJ, Burdette JH. Precentral gyrus discrepancy in electronic versions of the Talairach atlas. NeuroImage. 2004;21: 450–455. doi:10.1016/j.neuroimage.2003.09.032
